# Reform of the Medical College of Ohio

**Published:** 1835

**Authors:** 


**SUPPLEMENT**

TO THE

### WESTERN JOURNAL

OF THE

### MEDICAL AND PHYSICAL SCIENCES:

**FOR APRIL, MAY AND JUNE, 1835.**

### Reform of the Medical College of Ohio.

Within the last eighteen months, we have, from time to time, furnished our readers with facts relative to the Medical College of Ohio; and propose, in this number, to give them an account of some recent efforts to improve the condition of that ill-fated institution.

Its Board of Trustees, eleven in number, are appointed by our General Assembly, every three years. From the time of the appointment of the first Board early in 1823, nearly the same persons were re-elected, and when new ones were now and then introduced, they were generally nominated by those already in place; so that the same *animus*, directed the policy of the College, through this long period of twelve years, and the class never but once rose to 130 pupils, and for seven or eight years, from ten to sixteen, were beneficiaries, admitted under a statutory provision. Even as late as last year, the class of *bona fide* pupils was so small, that five weeks after the course commenced, the professor of Surgery had sold but 64 tickets.

The term of service of the Board appointed in January 1832, expiring with the year 1834, the General Assembly thought fit to elect a Board, but two of whom had ever served before, the remainder consisting of equally respectable gentlemen, both of town and country. Their names were given in the last number of the Journal.

While the election of the Board was pending in the Legislature, the following petition originated in Cincinnati, and being signed by a large majority of our physicians, was sent into the country, where it received more than 100 additional names, among which we recognize, many of the most respectable physicians of the state.

### MEMORIAL,

### TO THE HONORABLE THE BOARD OF TRUSTEES OF THE MEDICAL COLLEGE OF OHIO.

Gentlemen:—We the undersigned, physicians of the state of Ohio, feeling deeply for the interests of the Medical College, which appears to be in a languishing condition, when compared with its sister institutions, would respectfully suggest to your honorable body, that a change might be effected in its faculty, which would increase its strength, and make it more acceptable to the profession in the Valley of the Mississippi, and consequently more useful as a school of Medical science. What that change ought to be, will remain for those who preside over it to determine; but unless something be done speedily, it seems to us that it will, ere long, pass into entire neglect.

*Cincinnati, February 9th*, 1835.

Cincinnati.—C. R. Cooper, Silas Reed, M. Flagg, William Mount, William Wood, V. C. Marshall, William Judkins, Charles Woodward, John T. Shotwell, Isaac Colby, William S. Ridgely, Landon C. Rives, Charles Higbee, Stephen Bonner, W. Raymond Sanderson, B. Franklin Williams, Israel S. Dodge, Ezra Read, B. F. Bedinger, Wm. Barnes, Wolcott Richards, James M. Mason, Samuel S. Houston, B. S. Lawson, Thomas S. Towler, G. Bailey, Joseph Gardner, James M. Ludlum. 28.

Ohio.—Peter Allen, D. W. Rhodes, I. A. Parker, John A. Gano, William Steele, Joshua Martin, A. H. Brower, Charles C. Haddock, Thomas C. Shreve, Samuel Underhill, B. Michener, Charles D. Daggett,* James Welch, R. T. Elson, A. C. Bruce, David Mills, John K. Finley, Joseph W. Patton, C. I. Ward, I. Moore, James R. Hughes, Joel Fithian, James M. Cary, L. W. Trask, John G. Stevens, George E. Conant, Edwin Smith, I. Clements, William M. Charters, E. Dillon, N. H. Cass, Ignatius O'Ferrall, A. I. Bennett, William S. Myres, I. Bubant, William Blackstone, W. E. Thomson, Peter Spink, Jesse Paramore, Pliny M. Crume, F. A. Cunningham, Joseph Cole, Thomas Richmond, John O'Ferrall, I. T. Teller, R. W. Stearns, William M. Murdock, Merchant W. Muxfong, W. S. Richards, Sylvester Spelman, William W. Bancroft, William Bushnell, A. Sutherland, H.

---

* Of the respectability of this memorialist there can be no doubt, as he received the honorary degree of Doctor of Medicine, at the late commencement of the College.

Reintzel, A. G. Miller, John Free, W. D. Slocam, William M. Deming, Washington Moorehead, John A. Turner, William F. Collum, John N. Wilson, Elijah Cooper, William McHenry, Issaac S. Perkins, Thornton Marshall, M. M. Doulee, I. S. Prescott, I. S. Wilson, William Bell, William I. Hunt, Charles Reed, Isaac Steese, Alexander Anderson, P. Beaugrand, Elijah F. Bryan, Greenleaf Norton, H. Stidger, Alfred Heacock, R. G. Huntington, I. I. Brice, D. Marble, Isaac Fisher, Elias Fisher, Thomas H. Gibson, William N. Luckey, James B. Finley, Edson B. Olds, E. Webb, A. T. Davis, T. Welch, Joseph K. Sparks, A. Brooke, Cyrus Falconer, Jacob Hittel, I. McMechan, John McCrackin, J. M. Dillon, I. W. Warfield, H. H. Mott, J. C. Bennett, Abel Underhill, I. V. C. Teller, I. F. Patterson, I. Boyce, H. A. Robinson, P. Wallace, A. Beasley. 108.

This memorial was sent into the country with the following circular attached to it:

### CIRCULAR.

*Cincinnati, February* 19, 1835.

To the Medical Profession of Ohio:

Gentlemen:—The above memorial embraces by far the larger portion of respectable physicians in Cincinnati and vicinity. Indeed, it becomes us to say, that but nine or ten at most, with the exception of the faculty of the Medical College of Ohio, have refused to sign it, and the reasons generally assigned for the refusal were entirely of a personal character; at the same time acknowledging the necessity of a change in the faculty of the institution. Three of the ablest professors in the school have declared *their belief* that reform is necessary, and one of them has said, that he saw nothing objectionable in the memorial, and that for his own part, he would have no hesitation in signing it. We have taken the liberty of sending this memorial to our professional brethren throughout the state of Ohio, for the purpose of procuring their names and their influence to aid us in effecting this great work of reform. We would thank our friends to whom this paper is sent, to circulate it among the profession in their town and neighborhood, and return it to us as early in March as may be convenient. We ask them especially not to delay its return.

Most respectfully, etc.,

C. R. COOPER,
S. REED,
M. FLAGG,
WM. MOUNT,
WM. WOOD.

Dr. Drake having been informed of this undertaking, addressed to some of the gentlemen engaged in it, the following letter, which was likewise sent out with the memorial:

*Cincinnati, February*, 17, 1835.

To Doctors, Cooper, Reed, Flagg, Mount and Wood:

Gentlemen:—I understand that you and some of your friends are engaged in circulating among the physicians of Ohio, a memorial to the trustees of our Medical College, requesting them to reorganize its faculty. I entirely concur with you in the necessity of that measure, and am pleased to find the profession in the city, at length, disposed to co-operate with that of the state, in efforts which cannot fail to be productive of public benefit.

But, from the great number of times that my name has been mentioned in connection with the Medical College, and from the false impressions made on the minds of many persons, both in and out of the profession, that the sole object of reform is to replace me in the faculty, I feel it due, both to you and myself, to say, in the most explicit terms, that if offered a chair, I should not accept, unless such extensive changes where made, as to create a prospect of immediate and permanent success. I am in fact, not a candidate for a professorship; and I desire that none of my friends may prosecute the object of reform, with the least reference to my accommodation; or go one step further than they would consider necessary, if I were not in existence.

As far as a feeling of personal regard towards me, may blend itself with the desire to raise the institution to respectability, I feel grateful; but must advise those friends in whom it may exist, that I cannot allow it to bring upon me an obligation to re-enter the school, unless a state of things should arise, that would make it consistent with my honor and interests to do so,—of which, I trust they would cheerfully permit me to be the judge.

I am, most respectfully, your obedient servant,

DAN. DRAKE.

These documents were sent to the Board about the time of their first meeting, which was on the 14th of April. At that meeting they appointed a committee of three of their most intelligent and respectable members, one of whom, Dr. Rigdon of Hamilton, was a physician, with instructions to inquire into the state of the faculty, and the means of improving its condition and character. The committee deeply impressed with the responsibility which had been thrown upon them, and influenced by an anxious desire to improve the condition of the school, issued the following circular.

*Cincinnati, April 14th,* 1835.

SIR:—The Board of Trustees of the MEDICAL COLLEGE OF OHIO, appointed at the last session of the Legislature, held their first meeting this day. The members, a majority of whom appearing for the first time in their present capacity, without the experience necessary to a proper fulfilment of their new duties, are anxious to avail themselves of the counsel and views of those, whose business and positions in life, are most likely to enable them to judge correctly. It has been thought advisable to consult particularly, the prominent members of the profession in Ohio, on this important question. A committee was, in obedience to these views, appointed.

The undersigned, composing this committee, beg leave to call your attention to the subject of the MEDICAL COLLEGE, and to request from you such opinions, views, and advice, as your engagements will permit you to offer. We would especially call your attention to two points.

1. To what cause is the actual depressed state of the Medical College attributable? Is the defect to be sought in the faculty, or in the Board of Trustees? Or does the want of success depend upon circumstances, over which neither have had control.

2. If in the faculty, what remedy would you propose, which, under existing circumstances, may be practicable?

As early an answer as possible to those queries, is requested, as the Board meet on the 14th of May, to make the necessary arrangements for the next session.

Viewing the Medical College as a state concern, in which every citizen is interested, and relying upon your co-operation in a case so intimately connected with the honorable profession of medicine, and of science generally,

We have the honor to be your obedient servants,

MORGAN NEVILLE.
JOHN C. WRIGHT,
LOAMI RIGDON.

This circular met, we understand, with candid and respectful answers, from more than 40 physicians, resident in various parts of the state; which replies with the memorial previously sent in, became the basis of a report made by the committee, at the next meeting of the Board, on the 20th of May, and by them accepted and ordered to be spread on their minutes. It was as follows:

The committee, to whom was referred the duty of inquiring into the practicability of procuring able professors "to fill the chairs of the Medical College of Ohio," have obeyed the spirit of the resolution, as they understand it.

Viewing the institution as exclusively a state concern, and believing that opinions ought to be consulted beyond the limits of Cincinnati, the committee addressed themselves to the medical gentlemen of the state generally, through the medium of circular letters. They have the gratification of being able to report, that their communications were received with respect; and the prompt replies which were made to them, bear the strongest evidence of the great interest which the whole profession take in the prosperity of the college.

Your committee submitted two questions for the consideration of those they addressed:—First, as to the probable cause of the depressed state of the institution; second, as to the remedy. Various views of the subject have been given in the answers which have been transmitted, and which are herewith presented; but when scrutinized, the general conclusion seems to be unanimous, that the languishing existence of the college is attributable to the dissentions of the individuals composing the faculty at different periods; and to the want of scientific reputation in the teachers. The hostile influence imputed to the visitation of the cholera has only been intimated in some of the communications from the city.

The remedy suggested is reform or revolution, more or less extensive, according to the individual views and feelings of the writers. The complaints made against the actual incumbents are much diversified: in part they may be based upon personal knowledge, but principally seem to be the result of impressions made upon students during the session, which being widely reported by them on their return to their homes, are thus made to pervade every part of the great valley. The objections, according to the chair against which they are made, may be comprised under the heads of: — defect of elocution and eloquence; want of system and method; too great reliance upon compiled lectures; and last, though not least, a want of social courtesy and accessibleness on the part of some of the faculty. The last complaint, however, the committee is gratified to state, has been mostly confined to verbal communications to them, and is by no means of general application. It is but just to say, that in all the unfavorable opinions given, Drs. Eberle and Cobb are never included. The devotion of the former to the cause of medical science, and his wide spread reputation through Europe, as well as at home, are acknowledged, as making him as ornamental as useful to our infant institution; the latter is considered as filling his chair advantgeously and pleasantly.

A school of mere science depends more for success upon the established reputation of the teachers than any thing else. It is happy for that seminary, which receives students of strong preconceived opinions of the powers of those they are to listen to. Young aspirants after professional education, are not to be considered as very able critics; when placed under the instruction of men, who, by their writings, eloquence, or long services as lecturers, are favorably known to the world, they visit the school to be improved, and they listen to be satisfied. The case is too often the reverse, where the teachers are of unestablished reputation, even with more than ordinary abilities. The student becomes a critic, and is frequently liable to carry away erroneous judgment and false conclusions. Your committee is of opinion, that no college can prosper, without being distinguished for scientific character; and the voluminous correspondence with which they have been favored, together with many verbal communications, leaves the conviction, that according to impressions abroad, this character is not sufficiently pervasive as regards the Medical College of Ohio. It seems to be considered, that the philosophy of medicine is not sufficiently studied: that there is a want of that enthusiasm, that *esprit du corps*, that ambition of professional fame, without which no young institution can struggle through the morning of existence into ultimate usefulness.

In conclusion, in fulfilling the main object of their appointment, viz: "inquiring into the practicability of procuring able professors for the college," they state, that sufficient time has not been allowed to extend their researches very widely. The existing professors not having thought proper to resign, they, of course, are to be considered before the Board, for selection. Dr. Drake is on the spot, but has not signified to the committee that he is a candidate; individually, however, they can say they have no doubt of his acceptance of a chair, under an organization which, in his opinion, might promise to be successful. An exertion has been made to induce Dr. Silliman to accept a situation; this distinguished gentleman, however, after some hesitation, has declined, at the same time recommending in his place, his associate, Dr. Sheppard, advantageously known through New England, as an eminent teacher of chemistry. In addition to this last gentleman, the names of the following physicians have been most favorably reported to your committee, as calculated to give a happy impulse to the Medical College. Dr. Fearn, of Alabama, Dr. Cross, of Kentucky, and Dr. McDowell, now of Cincinnati. Dr. Fearn possesses an enviable reputation as a surgeon, and being a popular man and sound scholar, is considered as likely to use a beneficial influence throughout the south west. Dr. Cross is described as original, impulsive, and energetic, and is a distinguished contributor to some of the medical journals in the East. He has been more than once honored by the prize, for essays composed for the purpose. He is now in France for improvement in his profession, and will return by the autumn. Dr. McDowell made a favorable *debut* in Philadelphia,* as a lecturer, and since in Lexington; and is viewed by his friends as highly gifted and of much promise. He is full of zeal and enthusiasm. The attention of the committee has also been directed to the high claims of Dr. John Locke, of this city. This gentleman is well known, and some of your committee view him as one of the most learned men in the Valley of the Mississippi. Drs. Slack and Whitman, have also been presented. These gentlemen were formerly connected with the college, and are well known. Dr. Rives is also highly recommended by many most respectable signatures.

The organization of the chairs may be considered as properly coming within the duties of the committee; that is a subject, however, of some difficulty and doubt. It ought to be fully discussed before any appointments are made, and if a change in name and number is advisable, the plan suggested by Dr. Drake in his communication, is respectfully submitted, as combining probably the greatest amount of advantages. The correspondence herewith presented is tendered as part of the report.

MORGAN NEVILLE.
J. C. WRIGHT.
L. RIGDON.

At their meeting on the 14th of April, the Board, also appointed another highly respectable fiscal committee, one member of which, Judge Burke, had already served for many years, in the former Board; and on the 20th of May, they likewise reported, and their report was accepted and ordered to be recorded. It reads as follows:

The committee, appointed on the 15th of April, "for the purpose of reporting on the situation of the college to the next meeting of the Board," have made sundry inquiries into the fiscal and other concerns of the institution.

From the best information obtained by the committee, it appears that the claims now existing against the college amount to about twenty-four hundred dollars.

The only source of revenue accruing to the college, is that which arises from sales at auction; and, by a law of the state, this source will cease in the month of June, 1836.

The best estimate which the committee are able to make of the extent of the revenue accruing to the institution during the period of

*Dr. McD. delivered four courses in Cincinnati, one in Lexington, and his last in Philad.

about twelve months, in which time only the right of the college to that revenue will exist, cannot exceed $400 per quarter, or a total amount of $1600—leaving about $800, with the accruing interest, entirely unprovided for.

The committee are unable to advise the Board as to the best method to be pursued, to affect an extinguishment of the claims that must continue to exist, upon the college after the exhaustion of all the means applicable to their payment.

They are led to believe that the institution must, in a great measure, exist upon its own resources, and not depend hereafter upon legislative aid to any great extent.

The large amounts of money heretofore applied to the uses of the college, contrasted with the evident want of success which has attended the institution, has, it is believed, produced a feeling of strong repugnance in the legislature of the state, to afford any further aid; particularly when the opinion generally entertained in that body, for some length of time, has been: that an institution dignified with the name of the "Medical College of Ohio," has but too clearly indicated an arena for the display of local jealousies.

The committee not having been favored by the late Board of Trustees with a schedule of the valuable effects belonging to the college, are unable to give any information as to their extent or cost.

The only paper which the committee have been able to procure touching this branch of the subject, is a report (politely furnished by the present secretary) made to the late Board of Trustees, by the librarian, bearing date the 1st March, 1834, by which it appears that the library then consisted of 1642 volumes.

The committee cannot but believe, that with a proper attention to the interests entrusted to their care—with a fixed determination to promote those interests—having a due regard for their own character and standing, and throwing aside all feelings of a personal nature,—it will be in the power of the present Board of Trustees to exalt the character of the Medical School to such an extent, that the institution will not only be relieved from the present pecuniary embarrassments, but eventually regain the good feeling and fostering care of the legislature.

WM. S. HATCH.
WM. BURKE.
WM. STEPHENSON.

Cincinnati, May 20th, 1835.

The two committees taken together, make a majority of the whole Board, and their reports exhibit perfect unanimity of opinion, as to the depressed state of the institution, and the necessity of reform.

By some oversight of the Board, the memorial and letters of the physicians of Ohio were not read in that body, with the single exception of Dr. Drake's letter, which being referred to by the committee, became as it were an integral part of their report, and may therefore with propriety be published in connection with it. It is as follows:

*Vine Street, May* 16*th*, 1835.

MESSRS. NEVILLE, WRIGHT, AND RIGDON:—

GENTLEMEN:—I have the honor to acknowledge the receipt of your circular of the 14th ult., requesting information and opinions relative to the causes of the depressed condition of the Medical College of Ohio, and the means of its elevation. All that I propose to say in reply will be included under two heads—Professorships and Professors.

I. PROFESSORSHIPS.

In every medical institution, the chief object should be the study of the structure and functions (healthy and morbid) of the human body. For this purpose Cincinnati offers great and increasing facilities; and every practicable effort should be made to render them available to students. To this end, the present chair of Anatomy and Physiology should be divided; for the subjects which it embraces are sufficiently extensive to occupy the time and talents of two professors even for a longer time than four months.

Special or purely descriptive Anatomy, is as much as any single man can travel over in one session; and General Anatomy, or the classification of the elementary tissues of the different organs, Pathological Anatomy, or an account of those tissues in a state of degeneracy from disease—and Physiology, or the laws of life, constituting the philosophy of organization, are even more than can be taught by one professor within the same period.

General or Pathological Anatomy are in a great degree the creations of the present century; and now constitute the chief objects of attraction in the celebrated schools of Paris; to which students of medicine and young physicians make visits from all parts of the civilized world. These branches, with Physiology, are the true and proper objects of a chair of the *Institutes;* for every thing besides them, which is usually taught from that chair, belongs to the Theory and Practice. Such a professorship, under the title of *General and Pathological Anatomy and Physiology*, filled by a competent man, could not fail to prove irresistably attractive, and exert on the prosperity of the school, the most beneficial influence.

Every medical school should have an anatomical and surgical demonstrator, who should superintend the manipulations of the students in the dissecting rooms, and direct them in the use of the knife, not merely in dissections, but in surgical operations, an experience which, of course, they cannot acquire by sitting on the benches of the amphitheatre. Indeed unless they handle and apply the instruments, themselves, they never can become expert operators, nor do credit to the school in which they are educated.

All who intend to graduate, should be required as a prerequsite to take a dissecting ticket for one session, and adduce to the faculty, the certificate of their teacher that they had made diligent use of their opportunities. Ten dollars would be a suitable price for this ticket; and if the student took it during the first year of his attendance, he should be allowed to dissect and operate in the second year without any other charge than that for subjects.

When either the Professor of "Special Anatomy" of "General and Pathological Anatomy and Physiology," or of Surgery, might be sick or otherwise necessarily absent, it should be the duty of the Demonstrator to supply his place; and when either of them might for a particular purpose want any limb or organ from his rooms, it should be forthcoming.

By thus placing four able men on Anatomy (healthy and morbid,) Physiology and Surgery, the whole would be taught in such perfection that the school would rise steadily and rapidly into distinction.

Medical Jurisprudence properly belongs to all the chairs; but still a special course of lectures might be delivered with advantage to the pupils. It would not answer however to create a distinct chair for this purpose, but a lectureship might be established and given to the Demonstrator of Anatomy and Surgery. Five dollars might be charged for the ticket; and every student who intends to graduate should be requested to attend one course, and if it should be in his first year, he should be allowed to attend the second gratis.

A Medical College should have a Chemical Experimenter, who in the absence of the professor should fill his place; assist him in difficult experiments; and receive such compensation as the professor might be able and willing to make. Such a subordinate office would every year make up a class for examinations on Chemistry and Pharmacy, and perform such little illustrative experiments, as his ingenuity might devise; thus promoting the study of Chemistry and acquiring for himself some compensation, made voluntarily by the more liberal or ambitious pupils.

Still further to increase his usefulness and his profits, he might be allowed the use of the Laboratory for lectures in the summer which would contribute to retain pupils in the city (a most desirable object) whether we refer to their own interests, as students, or to the advantages which the school may confer upon the community.

Botany, especially in a new country like ours is an important branch of Medical Education; but as it cannot be taught in winter it is almost entirely neglected. The trustees however may promote its study by establishing a lectureship which might be given to the chemical experimenter who under such a significant expression in favor of this interesting branch of natural science, would be able to make up a summer class; for which he would institute his own price (and which no pupil should be required to attend his lectures) many would be induced to remain in the city, and thus become better prepared for graduation.

Clinical or hospital practice should be divided among the professors of the Theory and practice, Surgery and Obstetrics according to the nature of the cases, and as they might serve to illustrate the rules of practice in those different departments.

Of other professorships I have nothing special to say and will proceed to submit the whole to the Board in a tabular form.

### Professorships.

1. Special Anatomy.
2. General and Pathological Anatomy and Physiology.
3. Surgery.
4. Obstetrics and the diseases peculiar to women and children.
5. Chemistry and Pharmacy.
6. Materia Medica.
7. Theory and Practice of Medicine.

### Sub-professorships.

1. Anatomical and Surgical Demonstrator, and Lecturer on Medical Jurisprudence.

2. Chemical Experimenter and Lecturer on Botany.

In the University of Pennsylvania, the tuition fees for two courses of lectures, one dissecting ticket and one hospital ticket amount to $260. In the Medical College of Ohio the corresponding expense on the plan just delineated embracing a course of Medical Jurisprudence not taught in Philadelphia would be $225 making a difference in favor of our institution of $35; which is more than enough to defray the expenses of a pupil on his journey from North Carolina a maritime Virginia to Cincinnati.

The difference in the price of tuition between our school and that of Lexington for two sessions including a dissecting ticket in the latter would it is true be five dollars against us, but for this small sum, the student would have the benefit of one, or if he chose two courses of Medical Jurisprudence and of nine teachers instead of eight.

### II. Professors.

Can the Professorships and Lectureships of which I have spoken, be so filled as to bring the institution into immediate favor with the profession, and speedily place it on the level of the most distinguished schools of the Union? They certainly can. But to do this, not more than *one-third* of the present incumbents should be retained. That they have not, *as a faculty*, acquired the confidence of the profession in the Valley of the Mississippi, will appear to the Board of Trustees from the following statements of the number of their pupils compared with the number in Lexington, through a series of years; or in other words, from their beginning:

TABLE.

| Years. | Cincinnati. | Lexington. |
|---|---|---|
| 1819 | 00 | 38 |
| '20 | 25 | 93 |
| '21 | 30 | 138 |
| '22 | 18 | 171 |
| '23 | 00 | 200 |
| '24 | 15 | 234 |
| '25 | 30 | 281 |
| '26 | 80 | 190 |
| '27 | 101 | 152 |
| '28 | 101 | 206 |
| '29 | 107 | 199 |
| '30 | ~~124~~ 123 | 210 |
| '31 | 131 | 215 |
| '32 | ~~72~~ 150 | 222 |
| 32 — '33 | ~~102~~ 82 | 262 |
| 33 — '34 | ~~83~~ 114 | 247 |
| 16 | 1019 | 3020 |

Of the Cincinnati pupils. an average of 12, from 1826 to 1833 inclusive, were beneficiaries, and properly should not be included. The first year of the Lexington School is not added in.

Thus it appears that our school has not averaged more than one-third of the number of pupils of the Lexington school; and that the disparity last year was even greater than the year before. The cause must certainly be sought for in the relative talents and reputation of the two faculties; for as to local and statistical circumstances, and the expenditure of public money on the *material* of instruction, ours has enjoyed many important advantages over that. Indeed the central situation of Cincinnati, the great facility of intercourse which exists between it and other places; the amount of its population, and the number of its hospital cases, render it the most eligible city in the West for a medical institution, (not perhaps even excepting Louisville,) and present it as second only to Philadelphia. This being the case, it will obviously be practicable for the Board of Trustees to obtain professors of eloquence and intellectual power, devoted to the honor of the profession, and inspired with a love of fame. When this is done it must rise into distinction, but till then will continue to languish.

I have the honor to be, most respectfully,

Your obedient servant,

DAN. DRAKE, M. D.

The president of the Board had, also, in his charge, another letter from Dr. Drake, now on file as a public document, which it is necessary to introduce in this place:

*Vine Street, May 19th*, 1835.

My Dear Sir,—I intended to have seen you before this time, to reply to your inquiry whether I would be willing to accept of a chair in the Medical College of Ohio, Dr. Moorhead being one of my colleagues; but indispensable engagements have arisen, and are likely to continue, till the meeting of the Board to-morrow.

I thought my mind definitively made up on the question you put to me, but felt it a duty to review the whole matter, and again, converse with my friends upon it, this I have done but all who have expressed themselves are against it. Thus, I am confirmed by the reiterated injunctions of those whom I am accustomed to consult, and feel constrained to say, that I am afraid to venture into the institution with that person. When, *ex necessitate*, I was associated with him in 1831–2, I carried myself in every official interview with whatever discretion, moderation, decorum and courtesy I possess; but in the spring of 1833, when he gave testimony to the commissioners appointed by the governor, he represented me as overbearing, unjust and tyranical. If such was my deportment then it would be bad hereafter; and the peace and dignity of the college require, that I should not come into it, in connection with one who is strongly predisposed to recognize in me those unamiable qualities.

I wish to say explicitely, however, that *I do not and will not, stand in the way of a successful reorganization of the college*, an object which I have much at heart, and that should the Board reject me for Dr. Moorhead, but at the same time, improve the condition of the school, by the election of the able and efficient men, whose names I know to be before them, I will not only do all in my power to induce those gentlemen to accept, and enter the school without me, but I will, to the full interest of my humble means, do every thing to advance its interests, and augment its usefulness.

I am now too old to derive much advantage from a place in the college, as I was not very young when it was projected, 17 years ago; but I shall never be too old, to rejoice in its prosperity and honor.

This letter is private, but should you think, in any stage of the deliberations of the Board, that the reading of it might facilitate the very important measures of reform, which seem to me to be necessary, I beg you to regard it as a public document, and use it accordingly.

Very respectfully, your friend,

DAN. DRAKE.

JUDGE WRIGHT.

With the facts contained in these various documents before them, the Board, at their meeting of the 20th, came up to the task of reform and renovation. It had been expected by many persons, that before the time of this meeting, the professors would have resigned; several members of the Board had individually expressed, in society and even to professors, the opinion that they ought; the committee on the state of the faculty, seem to have entertained the same idea, when they wrote in their report, "the existing professors not having thought proper to resign are to be considered before the Board for selection;" and one of them, under a sense of delicate propriety, had indeed sent in his resignation, but was persuaded by his colleagues to withdraw it;—thus the Board found the professorships all filled, and had either to abandon the work of reform committed to them by the General Assembly or undertake the vacation of the chairs. In this dilemma, the chairman of the fiscal committee, offered a resolution declaring all the chairs vacant. The vote was taken and the motion lost. The friends of the weaker members of the faculty, were of course afraid that they could not be re-elected; and *Drs. Cobb, Smith, and Eberle, had entered into a compact, to remain in or go permanently out of the school together,** and therefore their friends were opposed to a general vacation of the professorships, lest in the failure of Dr. Smith on a re-election, Drs. Eberle and Cobb might not be at liberty to accept. Thus, as three members could prevent the vacation of the chairs, nothing was yet done.

The process of reform now took a new course, apparently that of declaring, *seriatim*, all the chairs vacant. Dr. Mitchell, with but one dissenting voice, and without any charge of incompetency, was displaced for Mr. Shepard

* We make this statement advisedly, and can prove it.

of Yale College. Dr. Pierson, professor of Mat. Med. in the equal absence of any allegation, was removed, by a unanimous vote; and Dr. Eberle, by a vote also unanimous was (with his own consent) transferred from the Theory and Practice to Mat. Med. and Dr. Drake elected to his place. The next vote was on Dr. Moorhead, professor of Obstetrics, when but five votes appeared against him, and his friends asked for reasons why he should be displaced, although two professors had just been dismissed without any reasons having been assigned! Was it impartial thus to change the rule of action in the progress of reorganization? And if it were done, should not the cases of Dr. Mitchell and Dr. Pierson have been reconsidered? or the justice of the Board defended, by a record on the minutes, of the defalcations and obliquities which rendered their expulsion necessary? But nothing of this kind was done, and the Board adjourned to meet again the succeeding night, having ordered the secretary to inform Dr. Drake and Mr. Shepard of their election.

Let us now ask, why send Dr. Drake a notice of his election and request an early answer, when his letter of the 19th declared that he should not accept, if Dr. Moorhead were retained? and, also, why notify Mr. Shepard of his appointment, when the Board had before them his letter to Dr. Drake, expressly, saying, "I am not willing to enter the school unless you are willing to go in?" Why elect two persons, and inform them of the fact, with a perfect knowledge that neither of them would accept? We confess ourselves unable to answer these questions. None of Dr. Drake's friends, with his knowledge or consent, had nominated him for a place; the committee on the state of the faculty expressly said that he was not a candidate, and his own letter published above, sets forth that he would not serve if Dr. Moorhead were retained, but would support a new and improved faculty without being a member of it.

In thus proposing to remain out of the school, and give it his humble support, as a physician of Ohio, provided it was made worthy of the state, did he not make all the sacrifice in his power? Was there any thing more he could have done? And should not this have defended him from an election and the clamor attempted to be raised on his non-acceptance? Did he stand in the way of a reorganization of the school? Is he censurable for the signal failure that has taken place? Is it he who has disappointed the hopes of the profession? For what purpose then, we once more inquire, send him and Mr. Shepard notices of their appointment, and permit one of the professors to communicate it for publication in the gazettes? The medical reader must answer this question for himself.

But what should be thought of the policy of attempting to combine Dr. Moorhead and Dr. Drake in the same faculty? All the members of the Board were acquainted with the fact, that from the first year of the emigration of Dr. Moorhead from Europe to Cincinnati, he had been Dr. Drake's unrelenting enemy; they all knew, that as late as the month of January last, he was one of the authors of a most bitter publication against Dr. Drake, entitled "Facts concisely stated," etc. Should they then have expected any thing but discord from placing the two in the same faculty? And yet, did not the committee, in their report above inserted, expressly recognise this as the first in point of rank, of the causes which had hitherto retarded the prosperity of the school? It is not necessary to the success of an institution, that the professors should be personal friends; they may even dislike each other—but their animosities must not generate quarrels, nor interfere with a hearty co-operation in every thing calculated to advance the interests of the school. Was it expected by the intelligent people of Cincinnati, and the physicians of the city and state, that Dr. Moorhead and Dr. Drake would be brought together in a new and improved faculty? Was it generally desired and recommended by them? We fearlessly declare it was not; and without having seen one of the letters written by physicians of the state to the trustees except our own, we confidently appeal to them to bear us out.

But Dr. Drake's refusal to serve with Dr. Moorhead, arose only in part from the fear of being involved by him in dissensions. He wanted confidence in Dr. Moorhead as a professor, and believes that our physicians did not, either in their letters or verbal communications to the Board, indicate him as one that *ought* to be retained. For six or seven years, he held the leading chair of the school, the Theory and Practice, during which it was so low, that the trustees, some of them his personal friends, found it necessary to transfer him to Obstetrics, one of the easiest chairs of a medical school; and even in that, he did not acquit himself with such ability as to rise above the second grade. He was, indeed, one of the three professors whom Drs. Eberle, Cobb and Smith declared, in the winter, must leave the school, and was, likewise, among those whom the committee on the state and character of the faculty inform us, the profession of Ohio, had *not* excepted from the proscription, which they considered as indispensable to the success of the school.

Thus, in thinking that two members of the Faculty, only, should be retained, Dr. Drake is with his brethren of the state, and although all the odium of cherishing this opinion may fall on him, he will find the burden light, as long as he is sustained, by knowing that he is with the respectable profession of the most populous state of the West.

Dr. Drake does not cherish of himself the opinion, that his entering the college could raise it, in opposition to a dead weight of incompetent colleagues. He did not choose to venture into the water to save it from drowning, and sink with it. He presumed himself at liberty, as a citizen of Ohio, to judge, while out of the school, of the propriety of entering it; and to make a decision, without having his right to do so, called in question by any of the trustees; or without having his refusal denounced by them, as dictation or obstinacy. In this, however, he was mistaken, as individual members of the Board, have felt themselves at liberty to censure him for not accepting; as though he had knocked at the door for admission, knowing who were already in, and when it was opened, had refused to enter.

But let us return to the proceedings of the Board, at their second meeting, which was on the evening of the next day after the first sitting. At this meeting the motion for vacating professor Moorhead's chair, was again made, when

Messrs. Hatch, Rigdon, Dunlavy, Carter and Fletcher, three of whom reside in the country, voted in the affirmative, and Messrs. Wright, Burke, Stephenson and Este, (the last elected the night before to supply a vacancy,) voted in the negative. Five to four, and of the four Mr. Stephenson declared the next day, that from what had been manifested as to the public sentiment of the profession, Dr. Moorhead ought not to be retained, and that he only voted for him, because no person was presented to supply his place, whom he thought eminently qualified. Thus he was, in fact, supported by three members only, two of whom Messrs. Burke and Este, had for several years been members of the old Board, and cherished the opinions and feelings of that body! Immediately after the results of this second vote were declared, a member who had been charged with Dr. Drake's reply, presented it to the Board, when it was read as follows:

*Vine Street, May*, 21*st*, 1835.

Sir,—I have the honor to acknowledge the receipt of your official note of this morning, announcing my appointment to the chair of the "Theory and Practice of Medicine" in the Medical College of Ohio.

In replying, I feel embarrassed by not having before me any official information, as to the persons who would be my colleagues; but report represents, that the honorable Board of trustees have retained two thirds of the old faculty. If this be the case, I shall feel it a duty to the profession and myself, to decline the appointment with which I have been honored; having a settled conviction, that the school cannot be raised from its present depression, if more than two of the professors, to whom its destinies have been confided, should be retained.

If the Board, in their wisdom, should think proper, to supersede two thirds of the late faculty, with new incumbents; and professor Moorhead should be among the displaced, they may consider me as having accepted, and that I shall stand ready to execute to Mr. Shepard a guaranty, of two thousand dollars per annum for three years, the condition proposed in my letter to professor Silliman, on which his consent to accept a professorship was obtained.

Most respectfully, your obedient servant,

DAN. DRAKE.

C. FLETCHER, Esq. *Sect'y.*

This reply, which seems on the face of it to be sufficiently clear and explicit, was followed by an adjournment *sine die;* and it was said by some members of the Board, that Dr. Drake and Mr. Shepard would probably both accept! It will be observed by a re-perusal of this answer, that the chief condition which it sets forth is, that two only of the faculty should be retained, and these were understood, to be Drs. Eberle and Cobb. It does not ask the trustees to make up the faculty of men nominated to them by Dr. Drake. It does not *require* that the professors just named should be retained. And still, *some* of the members of the Board, have promulgated, in society that Dr. Drake, would not enter the school unless, the chairs were all filled by his dictation. Had he seen proper to designate the persons with whom only he would serve, he had a legal and social right to do so, without offence to the trustees; but he did not, he merely indicated, that he could not successfully aid in raising the school from its state of depression, if more than two of the old professors were retained, and Dr. Moorhead should not be among the number dispensed with. For this it is, that his conduct is daily assailed in the streets by two or three members of the Board. We should think that an experience of twelve years, might have taught the Board, that other means than the abuse of Dr. Drake, are necessary to the elevation of the school. He had defended himself against misrepresentations through that long period, and with all the freshness of earlier years, proposes to do it now and hereafter. Two years ago, he made a successful defence against the whole Board, sustained by the whole faculty; and feels, but little solicitude in a conflict with a small minority, who have taken upon themselves the hopeless task of perpetuating abuses, to which two of them were parties in past times: He thinks the medical gentlemen of Ohio, (and even himself) as good judges of what is necessary for the school, as two or three gentlemen not of the medical profession, however respectable they may be as members of society: He believes that no medical college can flourish, without the confidence and support of those who are to direct the choice of schools for their pupils: He has been so long in the profession, that he feels it a duty, *when circumstances created by others* call him out, to speak faithfully and fearlessly: He respects his profession, and will not, knowingly, compromit its interests and dignity: They who violate the high trusts confided to them by the state, will, as heretofore, be exposed; and they who expect to silence him by raising a clamor, will be disappointed.

Dr. Smith was one of those, whom Dr. Drake regarded as of secondary rank in the school. He is speaking of the professor and not of the man. He has satisfied himself, that as a lecturer, Dr. Smith is inferior to Dr. Cobb and Dr. Eberle. He knows that Dr. Smith, although at the meredian of life, had not a reputation beyond the limits of Kentucky, where he had resided for some years, when he was elected into the college; and he has ascertained that there has been a falling off of nearly one-half, in the number of pupils from that state, since Dr. Smith came into the institution. In the session of 1831-2, Kentucky furnished 32 students, when the class was 131; the united classes of the two years since Dr. Smith came to the school, have amounted to 174, but the number of Kentucky students in the same two years, from the best accounts we have been able to obtain, (the class being so small last year, that no catalogue was published,) did not exceed 24 or 26, when it should have amounted to 42, to have been in the proportion of the year 1831-2. Even as far back as the session of 1827-8, the school had 20 Kentucky students, in a class not exceeding 100. Dr. Smith is not among the professors indicated by the physicians of Ohio, in their letters to the committee, as up to the mark; nor have the committee intimated any thing in his favor. Dr. Smith came into the school, when it was in a most unsettled condition, as an inquiry into its history and prospects was at the time pending in the legislature, he has expended no money on it, brought no pupils to it, and has no claims to be retained, if an abler and more influential incumbent can be found. Such an one, Dr. Fearn, of Alabama, was indicated to the Board, by the committee, and he was recommended among others by Dr. Shotwell, of this city, Dr. Cartwright, of Natchez, President Woods, of the University of Alabama, Mr. McKinley and Mr. Murphy, members of Congress from that state, Judge Crawford, of the Federal Court, Governor Gayle, and Professor Griscom, of New York — the whole of them in as high a strain of eulogy, as men of sense would allow themselves to utter. The place of residence of Dr. Fearn had, moreover, much to recommend it, as but few pupils have yet come from the South; and Dr. Drake did not, could not doubt, that he would be a great acquisition to the college, compared with the present incumbent, whose very name was unknown to the profession of Cincinnati, when he was elected. Dr. Drake is aware of the argument for the retention of Dr. Smith, founded on the personal disadvantages to that gentlemen of a removal; but are the personal inconveniences to Dr. Mitchell and Dr. Pierson, of less magnitude? Dr. Drake does not admit, that such considerations should weigh against the public good, and believes that the duty of Trustees has only been faithfully performed, when they have appointed the ablest men within their reach. He was anxious and willing to co-operate with the Board in obtaining men of the highest qualifications, and, with an inconsiderate zeal, offered pecuniary guarantees, endorsed by his friends, to four gentlemen, in the aggregate amount of thirty thousand dollars, to accept professorships, provided the Board should appoint them; but he did not make it a condition to his going into the school, that any of these eminent men should be chosen; he, on the contrary, told the Board that if they would reform the school, by the introduction of these or others, whose names were before them, he would, if Dr. Moorhead *must* be retained, remain out, and labor with other members of the profession for its elevation.

But let us go on to the called meeting of the Board on the 4th of June, inst. At this meeting an attempt was made to restore Dr. Eberle to the chair of Theory and Practice, which failed, and the Board adjourned to the next morning. Mr. Garrard of Cincinnati, was then elected to supply the place of Mr. Dunlavy of Lebanon, who had resigned, but the Board again adjourned, without accomplishing any thing. At night they still failed to retransfer Dr. Eberle from Materia Medica to the Theory and Practice, when they passed a resolution, delegating to the Faculty the power of assigning the duties of that chair to one or more of their own body, and adjourned for a week. The next day the Faculty met, and assigned the duty to Dr. Eberle, who is thus *professor* of Materia Medica, and *lecturer* on the Theory and Practice, the chair which he lately held; which is now vacant, and manifestly is expected to remain so for the next session, or the Board would not have directed the Faculty thus to dispose of the duties. Having done this, they adjourned for a week, to receive the answer of Mr. Shepard, who had previously declared that he would not accept unless Dr. Drake should do the same.

On the 12th inst. the Board again assembled, and had Mr. Shepard's reply, asking for information on several points, one of which was whether Dr. Drake would enter the college. The secretary was instructed to give the information required, and the Board adjourned for three weeks to await his final answer. This will carry on the disorganized state of the school ixto the month of July, when, should that gentleman finally decline, two of the chairs must still, as at the present time, remain vacant, with an apparently fixed determination on the part of some of the members of the Board, to suffer them to continue so, until resignations take place, and a thorough renovation can be effected.

Such is the history and present state of the project of reforming the Medical College:—a work suggested to the members of the legislature, by a large proportion of the physicians composing the Medical Convention, assembled last winter in Columbus;—prosecuted by the General Assembly, in the election of a new Board of Trustees;—declared to be indispensable, by one-hundred and thirty-six physicians of the state, in a memorial to the Trustees, *not met by a single opposing name;*—forcibly indicated, as necessary, by letters from more than forty physicians of the state, written in reply to a circular of a committee of the Board, which declares the school to be actually in a depressed condition; finally, pointed out as indispensable, by two committees of the Board, inserted in the first part of this article, the six members composing which committees, make up a majority of the whole Board—of which six, one-half, arrayed themselves against any further changes, than the dismissal of Dr. Pierson, one of the most learned and amiable of the Faculty, and Dr. Mitchell, acknowledged to be one of the ablest lecturers of the school! The result of the whole operation is this — the Board have published to the world, that the Faculty, it is true, are incompetent, but that the fewer incompetent men it contains, the better; and that, therefore, by reducing the number from six to four, they have improved its condition. As public journalists in the profession, we set forth this result for the information of students. EDITOR.

*Cincinnati, Ohio, June 24th,* 1835.

